# Reports of Death in the City of Buffalo, for the Month of Sept. 1861

**Published:** 1861-11

**Authors:** J. Whitaker

**Affiliations:** Health Physician


*Report of Deaths in the City of Buffalo for the month of August*, 1861.

*Reports of Death in the City of Buffalo, for the month of Sept.* 1861.

Accidents 15, Anemia 1, Ancurism 1, Apoplexy 1, Cancer of Breast 1, Cholera Infantum 41, Cholera Morbus 2, Concussion of the Brain 1, Consumption 19, Convulsions 20, Croup 1, Debility 4, Delirium Tremens 2, Dentition 2, Diarrhœa 8, Disease of the Brain 5, Disease of the Heart 3, Disease of the Liver 1, Disease of the Spine 1, Diphtheria 1, Dropsy 1, Dropsy Abdominal 1, Dropsy of the Brain 1, Dysentery 24, Fever Intermittent 1, Fever Puerperal Fever Scarlet 6, Fever Typhoid 1, Fever Typhus 1, Hemorrhage from Lungs 1, Hemorrhage from Womb 1, Inflammation of the Bowels 2, Inflammation of the Brain 1, Inflammation of the Lungs 2, Inflammation of the Lungs, Typhoid 2, Inflammation of the Peritoneum 1, Intemperance 3, Marasmus 1, Old Age 1, Premature Birth 1, Whooping Cough 13, unknown 6, Total 202. Still Born 9. One year and under 73. Between one year and twenty, 66. Between twenty and fifty 42. Over fifty 16. Ages unknown 5. Males 112. Females 88. Not given 2. Total 202.

J. WHITAKER, Health Physician.

